# Evaporative electron cooling in asymmetric double barrier semiconductor heterostructures

**DOI:** 10.1038/s41467-019-12488-9

**Published:** 2019-10-03

**Authors:** Aymen Yangui, Marc Bescond, Tifei Yan, Naomi Nagai, Kazuhiko Hirakawa

**Affiliations:** 1 Institute of Industrial Science, University of Tokyo, 4-6-1 Komaba, Meguro-ku, Tokyo, 153-8505 Japan; 2 LIMMS/CNRS-IIS, UMI 2820, 4-6-1 Komaba, Meguro-ku, Tokyo, 153-8505 Japan; 3 Institute for Nano Quantum Information Electronics, University of Tokyo, 4-6-1 Komaba, Meguro-ku, Tokyo, 153-8505 Japan

**Keywords:** Electrical and electronic engineering, Electronic properties and materials, Electronic devices, Semiconductors

## Abstract

Rapid progress in high-speed, densely packed electronic/photonic devices has brought unprecedented benefits to our society. However, this technology trend has in reverse led to a tremendous increase in heat dissipation, which degrades device performance and lifetimes. The scientific and technological challenge henceforth lies in efficient cooling of such high-performance devices. Here, we report on evaporative electron cooling in asymmetric Aluminum Gallium Arsenide/Gallium Arsenide (AlGaAs/GaAs) double barrier heterostructures. Electron temperature, *T*_e_, in the quantum well (QW) and that in the electrodes are determined from photoluminescence measurements. At 300 K, *T*_e_ in the QW is gradually decreased down to 250 K as the bias voltage is increased up to the maximum resonant tunneling condition, whereas *T*_e_ in the electrode remains unchanged. This behavior is explained in term of the evaporative cooling process and is quantitatively described by the quantum transport theory.

## Abstract (web summary)

Designing efficient integrated cooling solutions by controlling heat management in nanodevices remains a challenge. Here, the authors propose evaporative electron cooling in the AlGaAs/GaAs double barrier heterostructures quantum well achieving up to 50 K electron temperature reduction at 300 K.

## Introduction

Nanoscale miniaturization of semiconductor devices has enabled ultrahigh-density integration and ultrafast operation of transistors and optoelectronic devices. A significant and sustained upward trend of nanoscale miniaturization is expected for the coming years owing to tremendous growth of the so-called “new communication and information technologies”. However, this downscaling of devices has brought technological issues. High electric fields in such nanoscale devices generate hot carriers, which transfer their kinetic energies to the lattice, leading to the formation of hot spots^1–3^. The on-chip power density now exceeds 100 W cm^−2^ ^2,4^ leading to lattice temperature above 400 K^5^. These self-heating effects result in significant reduction in performances^6^ and lifetimes of the devices. Moreover, the refrigeration of the entire systems is extremely power consuming^3,7,8^. Accordingly, efficient integrated cooling solutions are listed in the International Roadmap for Devices and Systems as the top five long-term key challenges to address^9^. The engineering of efficient cooling is then one of the major scientific, technological, and environmental tasks in a context of energy resource shortage^10,11^.

The most commonly used solid-state refrigeration is based on the thermoelectric Peltier effect^12^. In the thermoelectric regime, electrons frequently experience scattering, leading to the degradation in the thermoelectric power factor *S*^2^*σ*, where *S* and *σ* are the Seebeck coefficient and the electrical conductivity, respectively. Furthermore, the materials used to obtain efficient Peltier effect such as BiTe are not compatible with the standard semiconductor fabrication processes.

Another interesting mechanism for solid-state refrigeration is the thermionic cooling^13^. Electrons thermionically emitted from the cathode transfer their kinetic energies to the anode and give rise to refrigeration in the cathode^14^. In 1990’s, the idea of thermionic cooling revived and semiconductor heterostructure refrigerators based on this concept were investigated^15–20^. Lattice cooling by as much as 1 to 3 K was observed at 300 K by using a single or multiple barrier structures^21–25^.

An alternative approach to reduce the hotspot effect is to directly cool down electrons before electrons transfer their kinetic energy to the lattice. The aim of the present work is to demonstrate that a significant electron cooling as much as 50 K is possible in a semiconductor heterostructure operating at room temperature. The studied Aluminum Gallium Arsenide/Gallium Arsenide (AlGaAs/GaAs) asymmetric double-barrier heterostructure, which combines resonant tunneling and thermionic emission, was originally proposed by Chao et al. as a lattice cooler^26^. Here, we demonstrate that this heterostructure is also very efficient for cooling the electron system in the quantum well (QW). The electron temperature, *T*_e_, in the QW as well as *T*_e_ in the electrodes are determined from photoluminescence (PL) measurements. At 300 K, *T*_e_ in the QW is remarkably reduced by as much as 50 K as the bias voltage is increased up to the maximum resonant tunneling condition. This behavior is qualitatively explained in terms of the evaporative cooling process, which is well-known in the field of the cold atom physics^27^. In this work, we have implemented the concept of the evaporative cooling in a solid-state system, i.e., the semiconductor heterostructures, and observed a significant electron cooling as much as 50 K at 300 K. The observed cooling behavior is quantitatively confirmed by quantum transport calculations that self-consistently couples the non-equilibrium Green’s function (NEGF) formalism for electrons with the heat equation (see Supplementary Methods).

## Results

### Asymmetric double-barrier heterostructures

The samples used in the present work were grown by molecular beam epitaxy and prepared by growing successively on an n-type GaAs substrate, a 300-nm-thick n-GaAs emitter layer (Si: 1 × 10^17^ cm^−3^), a 5-nm-thick undoped GaAs layer, an undoped 15-nm-thick Al_0.4_Ga_0.6_As barrier (we call this barrier “the emitter barrier” hereafter), an undoped 4-nm-thick GaAs QW, an undoped 100-nm-thick Al_0.25_GaAs_0.75_As barrier (we call this barrier “the collector barrier” hereafter), and a 200-nm-thick n-GaAs collector layer (Si: 1 × 10^17^ cm^−3^). We used a rather thick emitter barrier (15 nm) to quantum-mechanically decouple the electronic states in the QW from those in the emitter electrode and separate the photoluminescence (PL) in the QW from that in the electrodes. Thinner barrier would also degrade the heat insulation between the electrons in the QW and those of the emitter, leading to a reduction of the electron cooling. The wafer was then patterned into 200 × 200 μm^2^ mesas by photolithography. 70/50-nm-thick AuGeNi/Au contacts were deposited on the front and back sides of the mesa. For optical measurements, we made a 50 × 50 μm^2^ window on the top of each mesa, where we deposited semi-transparent 5-nm-thick NiCr film to ensure the uniformity of the applied bias voltage. The wafer was finally annealed at 450 °C in Ar ambient for 30 s. For more detail, see the Supplementary Note 1 and the Supplementary Fig. 1.

**Fig. 1:**
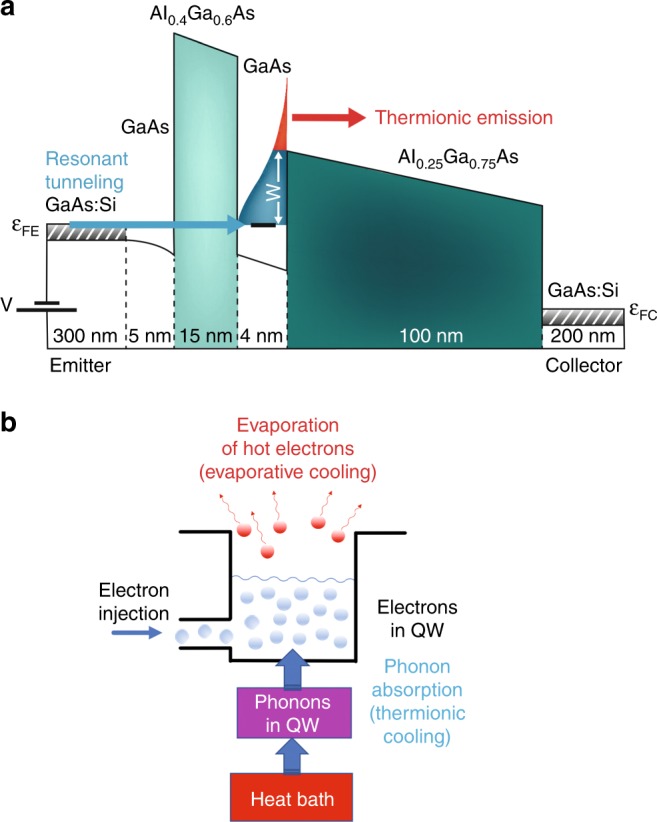
The asymmetric double-barrier heterostructure for evaporative electron cooling. **a** Band diagram of the asymmetric double-barrier heterostructure. Electron transport mechanism, i. e., the resonant tunneling through the thinner barrier (blue arrow) and the thermionic emission above the thicker barrier (red arrow), is schematically shown. **b** Schematic diagram for the heat flow in the electron-lattice system in the heterostructure. The concepts of the evaporative and thermionic cooling are schematically illustrated

Figure 1a illustrates the band diagram of the asymmetric double-barrier heterostructure. A positive voltage is applied to the collector electrode with respect to the emitter electrode. Electrons are injected from the emitter electrode into the quantized subband in the QW by resonant tunneling through the emitter barrier. Due to the very thick collector barrier, electrons injected into the QW cannot tunnel out to the collector electrode; instead, electrons whose kinetic energy is larger than the height of the collector barrier are removed by thermionic emission. This effect leads to the cutoff of the high-energy tail of the distribution function of electrons in the QW which then thermalized into a new quasi-equilibrium state at a lower temperature. This is the so-called evaporative cooling^28,29^.

Figure 1b schematically illustrates how the non-equilibrium electron and lattice systems in the QW interact with each other. The electron system is cooled through “evaporation” over the collector barrier. The colder electron system in the QW is in close contact with the warmer lattice system via electron–phonon interaction. As a result, the electron system is warmed up by the lattice system through phonon absorption, while the lattice system in the QW is cooled down by the electron system (“thermionic cooling”). Furthermore, since the QW is contacted by the electrode layers, there is a heat flow from the heat bath to the QW.

### Determination of the electron temperature

**Fig. 2:**
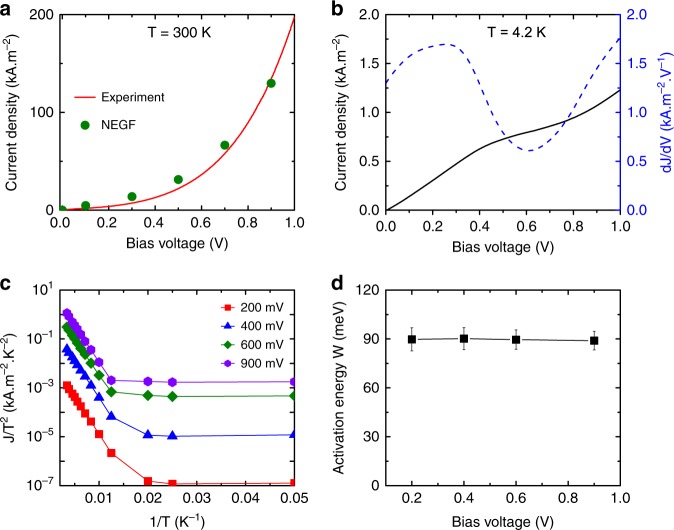
Electron transport measurements. **a** Current density *J* measured as a function of the bias voltage *V* at 300 K (red solid line). The green symbols are the calculated current densities by NEGF simulations. **b** *J*–*V* curve measured at 4.2 K (black solid line). The resonant tunneling shoulder can be clearly identified at around *V* ∼ 0.5 V. The dotted curve shows the differential conductance curve, d*I*/d*V*. **c** log(*J*/*T*^2^) is plotted as a function of 1/*T* at various *V*. **d** Thermal activation energy, *W*, determined from the *J*–*T* data shown in (**c**)

To characterize the sample structure, we first measured its current–voltage characteristics. Figure 2a plots the current density, *J*, measured as a function of the bias voltage, *V*, at 300 K. As seen in this figure, the *J*–*V* curve at 300 K is rather featureless and *J* gradually increases with increasing *V*. This *J*–*V* curve is perfectly reproduced by the NEGF quantum transport calculation. To gain more insight to the resonant tunneling electron injection, we also measured *J* at 4.2 K (see Fig. 2b). A kink due to the shut-off of the resonant tunneling process can be identified near 0.5 V, which is more clearly visible in the plot of d*I*/d*V*. This confirms the resonant tunneling injection of electrons into the QW. Figure 2c plots the temperature dependence of *J* measured at various *V*. An exponential decrease in *J* with decreasing *T* is observed for *T* > 100 K, whereas *J* becomes almost temperature-independent for *T* < 50 K, indicating that the current is carried by tunneling. The high-temperature behavior is dominated by the thermionic emission process^30^, whose magnitude is proportional to *T*^2^exp(-*W*/*k*_B_*T*). Here, *W* is the activation energy (see Fig. 1a) and *k*_B_ the Boltzmann constant. From the slope of the log(*J/T*^2^) vs 1/*T* plots at high temperatures, we estimated *W* (Fig. 2d). *W* does not depend on *V* and stays at around ~90 meV, which is consistent with the designed band structure.

**Fig. 3:**
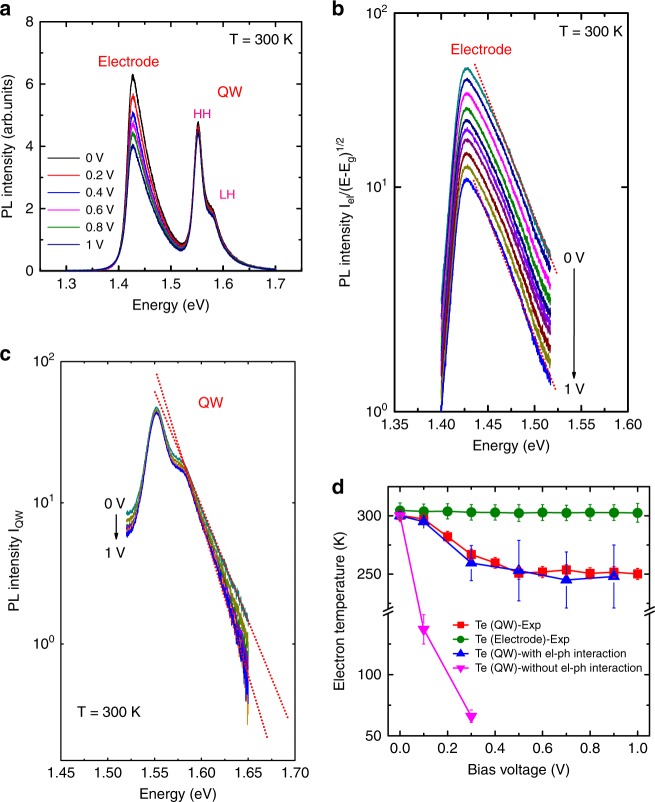
Electron temperature determined from photoluminescence measurements. **a** Photoluminescence (PL) spectra measured at 300 K for various bias voltages. Peaks corresponding to the recombination of electrons in the quantum well (QW) with heavy holes (HH) and light holes (LH) are also highlighted. **b** The plot of the PL intensity of the electrode, in a logarithmic scale, as a function of the photon energy shows that the slope of the high-energy tail is bias independent. The red dotted lines are eyeguides. **c** The intensity of the QW PL emission as a function of the photon energy shows that the high-energy tail of the PL spectra becomes steeper as the bias voltage is increased. **d** The electron temperatures in the QW (red squares) and electrode (green circles) determined from PL measurements are plotted as a function of the bias voltage. The blue triangles denote the electron temperature calculated by taking into account electron-phonon interaction, showing a good agreement with the experimental data (red squares). The pink triangles are the electron temperatures calculated by suppressing the electron-phonon interaction

Let us discuss the electron temperature, *T*_e_, in the QW. For this purpose, we carried out PL measurements at 300 K. The measurements were performed by using an excitation photon energy of 2.54 eV. This rather high photon energy is to reduce the penetration depth of the excitation light into GaAs (it is ~100 nm at 2.54 eV) and have a higher PL sensitivity in the shallow QW region. The excitation power was set to be 2 mW and the spot size was about 10 µm. Figure 3a plots the PL spectra measured at various *V* at 300 K. A PL peak is observed at ~1.43 eV, which results from the emission in the n-GaAs electrodes. A shaper PL peak is observed at 1.552 eV, together with a shoulder at 1.578 eV. The peak at 1.552 eV originates from the radiative recombination of the ground subband electrons in the QW with heavy holes (HH) and the shoulder at 1.578 eV is due to the electron-light hole (LH) emission.

Assuming the Maxwell–Boltzmann distribution for electrons, *T*_e_ in the QW as well as that in the electrodes were deduced from the high-energy tail of the PL spectra^31–33^. In the electrode regions, we have a three-dimensional density of states and the PL intensity can be approximately expressed as,

1 $$I_{{\mathrm{el}}} \propto \sqrt {h\nu - E_{\mathrm{g}}} {\mathrm{exp}}\left( { - \frac{{h\nu - E_{\mathrm{g}}}}{{k_{\mathrm{B}}T_{\mathrm{e}}}}} \right),$$

where *I*_el_ is the PL intensity of the electrode emission, *hν* the photon energy, *E*_g_ the bandgap of GaAs, and *k*_B_ is the Boltzmann constant. As for the PL from the QW, we have constant two-dimensional densities of states for electrons and holes. Then, we have

2 $$I_{{\mathrm{QW}}} \propto {\mathrm{exp}}\left( { - \frac{{h\nu - E_0}}{{k_{\mathrm{B}}T_{\mathrm{e}}}}} \right),$$

where *I*_QW_ is the PL intensity of the QW region, and *E*_0_ the energy difference between the electron-hole quantized ground subband. Using Eqs. (1) and (2), we replotted in Fig. 3b, c the PL intensities for the electrode and the QW, respectively. Figure 3b plots ${\mathrm{log}}[I_{{\mathrm{el}}}/\sqrt {h\nu - E_{\mathrm{g}}} ]$ as a function of *hν* for the electrode emission. The high-energy exponential thermal tail of the PL is clearly observed and *T*_e_ in the electrode is deduced from its slope. The determined *T*_e_ is ~305 K and remains almost unchanged when *V* is varied, as shown in Fig. 3d (green circles). Concerning the PL from the QW, we plot log*I*_QW_ as a function of *hν* in Fig. 3c and determine *T*_e_ from the slope of its high-energy tail. As seen in the figure, the slope becomes steeper as *V* is increased, reflecting the reduction in *T*_e_ in the QW. In Fig. 3d, the obtained *T*_e_ for the QW are plotted by red squares as a function of *V*. Note that *T*_e_ in the QW is reduced from room temperature by as much as 50 K when *V* = 0.5 V. For *V* above 0.5 V, *T*_e_ in the QW almost saturates.

As described before, the decrease in *T*_e_ in the QW results from evaporation of electrons^28,29,34^ and can be explained as follows; at *V* = 0, electrons in the QW are in the thermal equilibrium with those in the emitter and collector electrodes. The local net current vanishes and the electrons in the QW have the same thermal distribution as those in the electrodes, which are at room temperature. Applying a positive bias induces a net flow of electrons from the emitter to the collector. In the QW, the thermally excited carriers that have an energy higher than the collector barrier are driven outside the QW toward the collector. The electrons remaining in the QW are re-thermalized within a few tens of femtoseconds to a new quasi-equilibrium distribution at a lower temperature via electron–electron and electron–phonon scattering. The increase in *V* promotes the current flow, i.e., electron evaporation, until the maximal resonant tunneling is reached at *V* = 0.5 V. For *V* above 0.5 V, the subband in the QW goes below the conduction band edge in the emitter and the resonant tunneling injection should be shut off. However, as shown in Fig. 2b, the measured current does not decrease but it becomes almost constant. This is probably due to scattering-assisted injection of electrons from the emitter to the QW. Figure 3d shows an excellent agreement between experiment and quantum transport calculations. We note that electrons in the collector are heated up by the landing of hot electrons that escape from the QW. However, since the heat released by the hot electrons are very quickly redistributed and shared among very many electrons in the collector electrode, the increase in the electron temperature in the collector is expected to be much smaller (below 0.1 K). Furthermore, the surface metal electrode also helps good thermal anchoring of electrons in the collector electrode.

At this point, we would like to discuss interactions between the electron and the lattice systems. In polar materials such as GaAs, interactions between electrons and lattice are mainly mediated by polar optical (PO) phonons^35–37^. Electrons injected onto the QW state are then subject to PO phonon absorption. This transfer of energy from the lattice to the electron system warms up the electron system. To see this effect, we plot, in Fig. 3d, *T*_e_ in the QW obtained from quantum transport calculations in which the interaction between the electrons and the PO phonons in the QW is intentionally suppressed. It shows that the calculated reduction in *T*_e_ is significantly enhanced; for instance, *T*_e_ is 137 K at *V* = 0.1 V. For higher voltages (i.e. *V* superior than 0.5 V), the calculation of *T*_e_ cannot be numerically performed, because the present theoretical model assumes the acoustic phonon scattering to be elastic and, because of this reason, the quasi-localized state becomes inaccessible once it goes below the conduction band edge of the emitter.

### Efficiency of lattice cooling

**Fig. 4:**
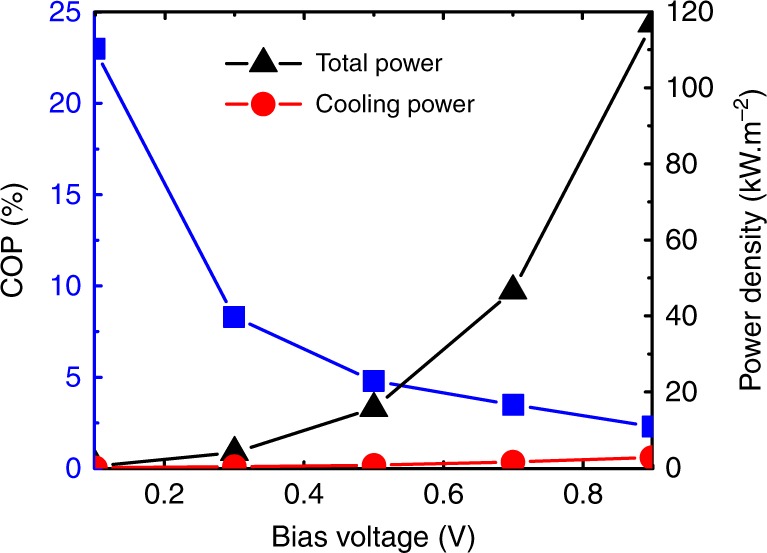
Cooling power density. The cooling power density (red circles) and the input electrical power density applied to the heterostructures (black triangles) as a function of the bias voltage. The coefficient of performance (COP) is then obtained from the ratio of these two quantities (blue squares)

Finally, let us make a comment on the lattice refrigeration effect. As previously described, phonon absorption by colder electrons in the QW cools lattice (thermionic cooling). Since the determination of the local lattice temperature in the QW is very challenging and out of the scope of the present paper, we want to make a theoretical estimation. Figure 4a shows the calculated cooling power density per unit area, *J*_Q_, as a function of *V*. *J*_Q_ is determined in NEGF from the spatial derivative of the electronic energy flow and defines the power transferred from the lattice to the electrons (see Supplementary Methods for more details)^38^. We see that *J*_Q_ increases with *V*. It goes from 111 W m^−2^ at 0.1 V to 2.7 kW m^−2^ at 0.9 V. However, the total input electrical power density, *JV*, applied to the heterostructure also strongly increases. A meaningful criterion is then given by the coefficient of performance, COP ≡ *J*_Q_/*JV*, which is also plotted in Fig. 4a. Interestingly, the COP shows a maximum at a low bias voltage (COP ∼ 23% for *V* = 0.1 V) and continuously decreases with increasing *V*. This feature results from the enhancement of the escape rate of electrons from the QW at high electric fields, hence reducing the interaction with phonons. Moreover, in this regime, the strong increase in the current component which directly goes above the collector barrier also contributes to the COP reduction. Figure 4 emphasizes the trade-off between a high COP, which is usually obtained near the equilibrium state, and a large cooling power, which is obtained at larger *V*.

## Discussion

Despite a good COP value, theory estimates a rather weak lattice temperature cooling, in the order of a few mK. The reason for such a small lattice cooling effect is threefold: the first is the difference in the specific heats of electrons and phonons. Indeed the specific heat of phonons in GaAs is, at room temperature, equal to 46 Jmol^−1^K^−1^, while the one for electrons is several orders of magnitude smaller^39^. The second is the high thermal conductance of the AlGaAs emitter and collector barriers. Since the thin QW is sandwiched between the emitter and the collector electrode layers, heat flow from the electrodes is significant. Third, we adopted a relatively low doping density in the electrode to reduce the peak width of the electrode PL (as shown in Fig. 3a).

For more significant lattice cooling, we need to increase the current density and realize a cooling power density of the order of 10^3^ W cm^−3^ in the QW. This value can be achieved by increasing the doping density in the emitter electrode and by reducing the thickness of the emitter barrier. Theoretical investigations reported in ref. ^40^ have shown that a structure with a higher doping level (10^18^ cm^−3^) in the emitter and a thinner emitter barrier (2.4 nm) can achieve a maximum COP above 100% (In the present cooling device structure, heat is removed from the QW and transferred to the collector region. Therefore, the COP estimated only for the QW region can exceed 100%) at *V* = 0.1 V, with a cooling power density of 2 × 10^2^ W cm^−2^. We are convinced that fully optimized structures will be able to locally refrigerate the lattice by a few tens degree celsius under typical operating conditions.

In this paper, we have investigated both experimentally and theoretically the cooling properties of AlGaAs/GaAs asymmetric double-barrier heterostructures. We have shown that electrons in the QW are refrigerated by the “evaporative cooling” process. Electron temperatures in the QW and in the electrodes were determined by PL measurements. We have found that, when operated at room temperature, *T*_e_ in the QW decreases down to 250 K with increasing the bias voltage up to the maximum resonant tunneling condition. The experimental results have been well explained by quantum transport theory. Furthermore, we have discussed the interplay between the electron and lattice cooling in the QW. The lattice system is refrigerated by the “thermionic cooling” process and its COP is in the order of 5–20%. These results make our heterostructure device promising for a comprehensive heat management in nanodevices.

## Methods

### Sample fabrication

A detailed description of the sample fabrication is provided in the Supplementary Notes 1 and 2 and the Supplementary Fig. 1.

### Electron temperature determination

The determination of electron temperatures from the fit of the high-energy tail of the PL spectra, assuming the Maxwell–Boltzmann distribution, has been well established and has often been used particularly for GaAs/AlGaAs heterostructures^31–33^. However, it is worth mentioning that the calculation of the electron temperature directly from the slope of high-energy tail of PL spectra relies on several approximations. Full details of the approximations used are provided in the Supplementary Note 3.

### Quantum transport code

Quantum transport code, including the calculations of current characteristics, electron temperatures, and coefficients of performance, can be found in the Supplementary Methods and in ref. ^40^.

## Supplementary information

Supplementary information

Peer Review File

Source Data file

## Data Availability

The data that support the plots within this paper and other findings of this study are available from the corresponding author upon reasonable request. The source data underlying Figs. 2a–d, 3a–d, and 4 are provided as a Source Data file.

## Code availability

The quantum transport code used for the calculations of current characteristics, electron temperatures and coefficients of performance are available from the corresponding author upon reasonable request.
