# Supplementary material for: Evaporative electron cooling in asymmetric double barrier semiconductor heterostructures
Source: Nat Commun. 2019 Oct 3;10:4504. doi: 10.1038/s41467-019-12488-9 (PMC6776518; doi:10.1038/s41467-019-12488-9)
Supplement: Supplementary file 1 — Supplementary information [file 41467_2019_12488_MOESM1_ESM.pdf]

# Supplementary Information

**Evaporative electron cooling in asymmetric double barrier semiconductor heterostructures**

Yangui et al.

## Supplementary Figures

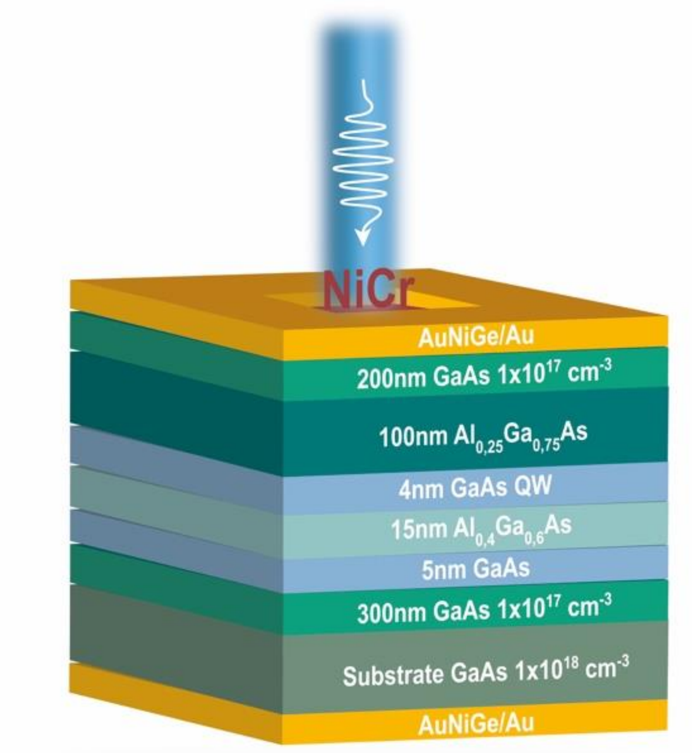


**Supplementary Figure 1:** Schematic illustration of the sample used in this study.

## Supplementary Notes

**Supplementary Note 1.** The Supplementary Figure 1 schematically represents the sample used in this work. The growth sequence is described in the main text. We note that the 5 nm-thick undoped GaAs layer below the emitter barrier is inserted to prevent the diffusion of Si donors into the thin $Al_{0.4}Ga_{0.6}As$ emitter barrier due to the segregation effect.

**Supplementary Note 2.** We used a rather thick emitter barrier (15 nm) to quantum-mechanically decouple the electronic states in the QW from those in the emitter electrode and separate the photoluminescence (PL) in the QW from that in the electrodes. When the emitter barrier was 2-nm-thick, as originally proposed by Chao et al.[1] the PL peak from the QW could not be resolved from that from the electrodes, because the electronic states in the QW were significantly hybridized with those in the emitter electrode.

**Supplementary Note 3.** The determination of electron temperatures from the fit of the high energy tail of the PL spectra, assuming the Maxwell-Boltzmann distribution, has been well established and has often been used particularly for GaAs/AlGaAs heterostructures (please see for instance refs [31-33] of the manuscript). However, it is worth mentioning that the calculation of the electron temperature directly from the slope of high-energy tail of PL spectra relies on several approximations:

- First of all, the slope of PL spectra reflects both electron and hole temperatures. However, holes, which have a heavier effective mass, are supposed to have a temperature closer to the lattice temperature, while electrons are supposed to be in a non-equilibrium state at a different temperature given by the slope of the PL spectra.[2] A good hint to assess this statement is given by the ratio $\alpha = m_e/(m_e+m_h)$. The smaller is $\alpha$, the closer is the slope of the PL to the electron temperature. In GaAs, $m_e$=0.67$m_0$ and $m_h$=0.45$m_0$, leading to $\alpha$=0.13. It has been shown that, when $\alpha$=0.05, the electron temperature calculated from the slope of the PL spectra is equal to the electron temperature extracted from the general expression of Planck's law with two temperatures with an accuracy of 0.05%.[3] Although $\alpha$ for GaAs is not as small as 0.05, we can still safely assume

that the impact of the hole component on slope of the PL spectra is negligible and that this PL technique provides a good assessment of the electron temperature.

- The second approximation comes from the fact that we assumed that the PL originates from the band-to-band transition and we neglected the excitonic emission effects. However, since exciton binding energy in GaAs is of the order of 5 meV,[4] we can safely neglect the excitonic effect in PL measurements at room temperature.

- The third main approximation lies in the energy-dependence of the absorption coefficient $A(E)$. Indeed, a more "rigorous" description of the PL spectrum includes the absorption coefficient as follows:[5]

$$I_{\mathrm{PL}}(E) \propto A(E) e^{-\frac{(E-E_{\mathrm{g}})}{k_{\mathrm{B}}T}}.$$

Approximating the high-energy PL tail by a "simple" exponential assumes that the absorption coefficient is energy-independent. Study from Ihara and co-workers have demonstrated that such assumption is indeed accurate in GaAs QWs at temperatures from 20 K to 220K.[6] This result should remain valid at room temperature, validating the use of our technique to prove the electron cooling phenomena in the GaAs QW.

In view of these explanations, the determination of the electron temperature based on the high-energy slope of the PL spectra is reliable to demonstrate the electron cooling by several tens of kelvins.

## Supplementary Methods

To simulate the electrical and thermal properties of the III-V heterostructure a self-consistent Schrödinger solver based on the Non-Equilibrium Green's Function (NEGF) formalism[7] was coupled with the heat equation.

**Electronic quantum transport.** The NEGF equations, which describe the electron transport, are expressed within the single-band effective mass Hamiltonian representing the Γ-valley of the conduction band of he III-V semiconductors. The considered structure being invariant in the plane perpendicular to the growth direction, Born-Von-Karman periodic boundary conditions are applied on the transverse wave vector component $k_t$ such that $k_t = n_{k_t} \times 2\pi/L_t$, with $L_t = 80$

nm and $n_{k_t}$ an integer indexing the transverse modes, whose degeneracy is equal to $\pi(2 \times n_k + 1)$.[8] In the following, we summarize the main features of the NEGF approach in matrix notation. We first define the retarded Green's function for each transverse mode $k_t$,

$$G^r_{k_t} = \left[(E - V_p)I - H_{k_t} - \Sigma^r_{L,k_t} - \Sigma^r_{R,k_t} - \Sigma^r_{S,k_t}\right]^{-1}, \tag{1}$$

Where $I$ is the identity matrix, $H_{k_t}$ represents the effective mass Hamiltonian for the transverse mode $k_t$ and $V_p$ is the electrostatic potential energy. $\Sigma^r_{L/R,k_t}$ and $\Sigma^r_{S,k_t}$ are the retarded self-energies for the left/right semi-inifinite device contacts and scattering mechanisms, respectively.

From the retarded Green's function, the lesser/greater Green's function is then obtained as

$$G^{\lessgtr}_{k_t} = G^r_{k_t}\left(\Sigma^{\lessgtr}_{L,k_t} + \Sigma^{\lessgtr}_{R,k_t} + \Sigma^{\lessgtr}_{S,k_t}\right)G^{r\dagger}_{k_t}, \tag{2}$$

Where the $\Sigma^{\lessgtr}$ are the lesser/greater self-energies, related to their retarded counterpart by

$$\Sigma^r = \frac{1}{2}[\Sigma^> - \Sigma^<]. \tag{3}$$

Only acoustic- and polar optical-phonon interactions are considered since non-polar optical phonons are found to be negligible in such III-V materials. Interactions self-energies are calculated within the self-consistent Born approximation (SCBA).[9] In our approach, the acoustic and polar optical-phonon baths locally follow a Bose-Einstein distribution, and are therefore assumed at equilibrium. On the other hand, acoustic and optical phonons can not be assumed at equilibrium with respect to each other, as the net anharmonic decay of optical phonons into acoustic, driven by an unbalance in their respective energy densities, plays a fundamental role in the thermal transport. This is taken into account in our model by defining, at each position of the domain, two different temperatures, $T_{AC}$ and $T_{PO}$ for the acoustic and polar optical-phonons, respectively. As detailed in the next subsection, these temperatures are self-consistently computed by coupling the electron transport equations with the heat equation.

Interactions with acousitic phonons are assumed to be elastic and described within the deformation potential approach. For a given wave vector $k_t$, the SCBA self-energy for acoustic phonons is:[10]

$$\Sigma^{\lessgtr}_{AC}(j,j;E) = \sum_{k'_t} \pi(2n_{k'_t} + 1)\frac{\Xi^2 k_B T_{AC}(j)}{\rho u_s^2} G^{\lessgtr}_{k'_t}(j,j;E), \tag{4}$$

where $\Xi$ is the deformation potential, $\rho$ is the mass density and $u_s$ is the sound velocity. In equation (4) index $j$ indicates the position along the growth discretized axis. Interactions with acoustic phonons are here assumed to be local and we then consider only the diagonal part of the

Green's function. Such approximation has been demonstrated to be valid for acoustic phonons down to very low temperatures.[11]

Interactions with polar optical (PO) phonons are also treated locally based on a scattering self-energy recently proposed as an effective description of their long range interactions.[12] For a given wave vector $k_t$, it can be shown that:

$$\Sigma^{\lessgtr}_{PO,k_t}(j,j;E) = \frac{\lambda M^2}{2\pi S}\sum_{k_t'}\Big[(n_L(j)+1)G^{\lessgtr}_{k_t'}(j,j;E\pm\hbar\omega_L) + (n_L(j))G^{\lessgtr}_{k_t'}(j,j;E\mp\hbar\omega_L)\Big] \times \int_{\pi/L_t}^{\pi}\frac{\pi(2n_{k_t'}+1)}{\sqrt{(k_t-k_t'\cos\theta)^2+(k_t'\sin\theta)^2}}d\theta, \quad (5)$$

with $(n_L(j) = (e^{(\hbar\omega_L)/(k_BT_{PO}(j))}-1)^{-1}$, $M^2 = 2\pi\hbar\omega_L e^2(\frac{1}{\epsilon_\infty}-\frac{1}{\epsilon_0})$, $\theta$ is the angle between $k_t$ and $k_t'$, $S=\pi L_t^2$, and $\omega_L = 35$ meV. $M$ is the Fröhlich factor in which $\epsilon_\infty$ and $\epsilon_0$ represent the static and high frequency dielectric permittivities, respectvely. Finally, $\lambda$ is a scaling factor which takes into account for the diagonal approximation. The value $\lambda = 6$ used in this paper has been obtained within the comprehensive and physically based analytical model proposed in [6].

The total phonon self-energy $\Sigma^{\lessgtr}_{S,k_t}$ for a given mode $k_t$ is then

$$\Sigma^{\lessgtr}_{S,k_t} = \Sigma^{\lessgtr}_{AC} + \Sigma^{\lessgtr}_{PO,k_t}. \quad (6)$$

Once the lesser/greater Green's function $G^{\lessgtr}_{k_t}$ of each mode $k_t$ is determined, physical quantities can be straightforwardly calculated.[9] The electron density $n$ along the transport direction reads:

$$n_j = -2\times\frac{i}{2\pi}\sum_{k_t}\pi\left(2n_{k_t}+1\right)\int_{-\infty}^{+\infty}G^{<}_{k_t}(j,j;E)dE = -i\int_{-\infty}^{+\infty}G^{<}(j,j;E)dE, \quad (7)$$

With $G^{<}(j,j;E) = \sum_{k_t}(2n_{k_t}+1)G^{<}_{k_t}(j,j;E)$. The carrier current density (in A.m$^{-2}$) flowing from position $j$ to $j+1$ along the growth direction is calculated from the off-diagonal elements $(j,j+1)$ of $G^{<}(i,j;E)$ as

$$J_{j\to j+1} = \int_{-\infty}^{+\infty}dE\,\frac{e}{\hbar}\left[H_{j,j+1}G^{<}(j+1,j;E) - G^{<}(j,j+1;E)H_{j+1,j}\right], \quad (8)$$

$$= \int_{-\infty}^{+\infty}\mathcal{J}_{j\to j+1}(E)dE, \quad (9)$$

where $H_{j,j+1}$ corresponds to the nearest neighbors hopping terms in the discretized tight-binding like Hamiltonian and $\mathcal{J}_{j\to j+1}(E)$ is the current density spectrum (in A.(m$^2$.eV)$^{-1}$). From equation (9) we can deduce the corresponding electronic energy current:[13]

$$J^E_{j\to j+1} = \int_{-\infty}^{+\infty} E\mathcal{J}_{j\to j+1}(E)dE. \quad (10)$$

The equations (1)-(6) are solved self-consistently using the recursive algorithm[14] until the criteria of convergence for both electron density and carrier current density are reached. The potential energy $V_p$ is self-consistently determined by nonlinearly coupling the transprot equations (1)-(6) with the Poisson equation through the electron density.

**Heat transport model and calculation of the coefficient of performance (COP).** We compute the lattice temperature by solving the 1D heat equation along the heterostructures growth direction (x). The discretized heat equation on the site $j$ reads:

$$\left[-\frac{\partial}{\partial x}[\kappa_{th}(x)]\frac{\partial}{\partial x}T_{AC}(x)\right]_j = Q_j, \quad (11)$$

where $\kappa_{th}$ is the thermal conductivity. It is set to be equal to the value for bulk GaAs (46 W.K$^{-1}$.m$^{-1}$) throughout the structure, except for the QW region, where it is set to be 4 W.K$^{-1}$.m$^{-1}$ in order to take into account the thermal resistance induced by diffusive phonon scattering at the heterointerfaces.[15] A small deviation of the value has no significant impact in the calculated electron temperature. The temperature entering the heat equation is the one associated to the acoustic phonons, since they have a larger velocity than their polar optical counterparts and are mainly responsible of the heat transport.[16] Left and right reservoirs are assumed to be at the thermodynamic equilibrium at $T_{AC}$= 300 K. This value is set by enforcing the Dirichlet's boundary conditions at the left and right contact. $Q_j$ is the volumetric source term which corresponds to the heat power density (in W.m$^{-3}$) generated by the electron-phonon interactions. In the framework of the previously described electron transport formalism, it can be computed as:[13]

$$Q_j = -\nabla_j \cdot J^E. \quad (12)$$

A negative value of $Q_j$ corresponds to an energy transfer from the lattice to electrons, while a positive one describes the reverse phenomenon. In the absence of electron-phonon interactions, $Q_j = 0$. By integrating over the position $j$ the negative part of $Q_j$, we can deduce $J_Q$, the cooling

power density. The COP is finally determined as the ratio of $J_Q$ by the applied power density, ($P_{Elec} = J \times V$).

From a physical point of view, electrons loose or increase their energy by scattering with polar optical-phonons. In turn, optical phonons decay into acoustic phonon modes, which sustain the thermal energy propagation long the device. In stationnary conditions, the power transfer from optical to acoustic phonons must be equal to the heat power density $Q_j$ defined above. Within a relaxation time approximation, we can thus write:

$$\frac{(T_{PO}(j) - T_{AC}(j))C_{PO}}{\tau_{PO\to AC}} = Q_j, \quad (13)$$

where $\tau_{PO\to AC}$ is the relaxation time of polar optical phonons into acoustic phonons ($\tau_{PO\to AC} = 4.16 \times 10^{-12}$ s)[17] and $C_{PO}$ is the thermal capacitance of the polar optical phonons per unit volume ($C_{PO} = 1{,}72.10^6$ J.(m$^3$.K)$^{-1}$.[17] The numerator of the left side expresses the average energy per unit volume exchanged between the polar optical and acoustic phonons baths in an interval $\tau_{PO\to AC}$. Equation (13) allows us to compute $T_{PO}(j)$ from the knowledge of $Q_j$ and $T_{AC}(j)$.

The computed values of $T_{AC}$ and $T_{PO}$ are injected in equations (4) and (5) respectively. This establishes the coupling between the heat equation and the electron transport equations. The heat equation is iteratively solved together with the transport equations and the Poisson equation, until a global self-consistency is achieved.

**Local electron temperature.** Once the convergence of those free equations is reached, the local electronic temperature is calculated in a post-processing treatment based on the virtual probe approach.[18] Such a method can determine the electronic temperature and electrochemical potential by cancelling the particle and energy currents between a floating probe and the nanostructure. The probe is then in local thermodynamic equilibrium with the non-equilibrium structure. Formally, we consider a thermoelectric probe at the position *j* along the *x*-axis defined by the following self-energy (similar to the Büttiker probes:[19]

$$\Sigma^{>}(j;E) = -i\left[1 - f_{FD}\left(E, \mu_j, T_j^e\right)\right] LDOS(j;E)\nu_{coup}, \quad (14)$$

$$\Sigma^{<}(j;E) = i f_{FD}\left(E, \mu_j, T_j^e\right) LDOS(j;E)\nu_{coup}, \quad (15)$$

where $f_{FD}$ is the Fermi-Dirac distribution of the electrons in the probe, $\mu_j$ and $T_j^e$ are respectively the local electrochemical potential and electronic temperature at the position $j$ ; $LDOS(j;E) =$

$i\,\frac{[G^{>}(j,j;E)-G^{<}(j,j;E)]}{2\pi}$ is the local density of states of the probe (taken equal to the one of the device) and $\nu_{coup}$ is the energy independent coupling strength between the probe and the system. In the considered case, the exact value of $\nu_{coup}$ is not important, as it will cancel out in the following computations. By enforcing the simultaneous cancellation of the electron charge and energy currents between the device and the probe, we obtain a system of two coupled nonlinear equations with the unknowns $\mu_j$ and $T_j^e$:

$$\Delta J(j) = \int_{-\infty}^{E_{Lim}} \Sigma^{>}(j;E)\, G^{<}(j,j;E)dE - \int_{-\infty}^{E_{Lim}} G^{>}(j,j;E)\Sigma^{<}(j;E)\, dE = 0, \quad (16)$$

$$\Delta J^E(j) = \int_{-\infty}^{E_{Lim}} E\, \Sigma^{>}(j;E)\, G^{<}(j,j;E)dE - \int_{-\infty}^{E_{Lim}} E G^{>}(j,j;E)\Sigma^{<}(j;E)\, dE = 0. \quad (17)$$

The upper energy integration limit $E_{Lim}$ results from the fact that PL measures the energy distribution of electrons that relax their energy in the QW and recombine with holes. In other words, the PL measurements do not see electrons that ballistically fly over the collector barrier.

In order to stay as faithful as possible to the experimental conditions, we therefore theoretically consider electrons whose energy is lower than the top of the emitter barrier. Since the electron temperature depends on $E_{Lim}$, we used four different $E_{Lim}$ and took an average of the obtained values. Moreover, the number of hot electrons which are transmitted from the emitter towards the collector by direct tunneling increases with the applied bias. The choice of $E_{Lim}$ is then more impacting the electron temperature at high biases. This is the reason why we have larger error bars for higher biases in Fig.3-d of the manuscript.

We note that the assumption of the contacts at 300K corresponds to massive contacts with sufficient electron thermalization. In the present study, we considered a long enough collector region (200 nm) to allow electrons to be entirely thermalized. In this configuration, the heat power density $Q_j$ integrated over the whole device must be equal to the applied power density, $P_{Elec} = J \times V$. In the calculations, this point has been verified systematically for each applied bias. We should also note that reducing the collector length to 100 nm does not affect the calculated electron temperatures in the quantum well.

The system is iteratively solved at each position $j$ through a Newton-Raphson algorithm.[20] A full description of the model can be found in Ref. [21].

## Supplementary References


1. Chao KA, Larsson M, Mal'shukov AG. Room-temperature semiconductor heterostructure refrigeration. *Appl. Phys. Lett.* **87**, 022103 (2005).
2. Shah J, Lin C, Leheny R, DiGiovanni A. Pump wavelength dependence of hot electron temperature in GaAs. *Solid. State. Commun.* **18**, 487-489 (1976).
3. Gibelli F, Lombez L, Guillemoles J-F. Two carrier temperatures non-equilibrium generalized Planck law for semiconductors. *Phys. B. Condens. Matter.* **498**, 7-14 (2016).
4. Tarucha S, Okamoto H, Iwasa Y, Miura N. Exciton binding energy in GaAs quantum wells deduced from magneto-optical absorption measurement. *Solid. State. Commun.* **52**, 815-819 (1984).
5. Cardona M, Peter YY. *Fundamentals of semiconductors*. Springer: Berlin (2005).
6. Ihara T, *et al.* Thermal-equilibrium relation between the optical emission and absorption spectra of a doped semiconductor quantum well. *Phys. Rev. B.* **80**, 033307 (2009).
7. Haug H, Jauho A-P. *Quantum kinetics in transport and optics of semiconductors*. Springer (2008).
8. Cavassilas N, Michelini F, Bescond M. Modeling of nanoscale solar cells: The Green's function formalism. *J. Renew. Sustain. Energy.* **6**, 011203 (2013).
9. Jin S, Park YJ, Min HS. A three-dimensional simulation of quantum transport in silicon nanowire transistor in the presence of electron-phonon interactions. *J. Appl. Phys.* **99**, 123719 (2006).
10. Jacoboni C, Reggiani L. The Monte Carlo method for the solution of charge transport in semiconductors with applications to covalent materials. *Rev Mod Phys* **55**, 645-705 (1983).
11. Bescond M, Carrillo-Nuñez H, Berrada S, Cavassilas N, Lannoo M. Size and temperature dependence of the electron–phonon scattering by donors in nanowire transistors. *Solid. State. Electron.* **122**, 1-7 (2016).
12. Moussavou M, Lannoo M, Cavassilas N, Logoteta D, Bescond M. Physically based Diagonal Treatment of the Self-Energy of Polar Optical Phonons: Performance Assessment of III-V Double-Gate Transistors. *Phys. Rev. Appl.* **10**, 064023 (2018).
13. Lake R, Datta S. Nonequilibrium Green's-function method applied to double-barrier resonant-tunneling diodes. *Phys. Rev. B.* **45**, 6670-6685 (1992).
14. Sancho MPL, Sancho JML, Rubio J. Quick iterative scheme for the calculation of transfer matrices: application to Mo (100). *J. Phys. F. Met. Phys.* **14**, 1205 (1984).
15. Luckyanova MN, *et al.* Anisotropy of the Thermal Conductivity in GaAs/AlAs Superlattices. *Nano. Lett.* **13**, 3973-3977 (2013).
16. Pop E, Sinha S, Goodson KE. Heat Generation and Transport in Nanometer-Scale Transistors. *Proc IEEE.* **94**, 1587-1601 (2006).
17. Tengfei L, Jivtesh G, Junichiro S, Keivan E, Gang C. Gallium arsenide thermal conductivity and optical phonon relaxation times from first-principles calculations. *EPL (Europhysics Letters).* **101**, 16001 (2013).
18. Shastry A, Stafford CA. Temperature and voltage measurement in quantum systems far from equilibrium. *Phys. Rev. B.* **94**, 155433 (2016).
19. Büttiker M. Role of quantum coherence in series resistors. *Phys. Rev. B.* **33**, 3020-3026 (1986).

20. Venugopal R, Paulsson M, Goasguen S, Datta S, Lundstrom MS. A simple quantum mechanical treatment of scattering in nanoscale transistors. *J. Appl. Phys*. **93**, 5613-5625 (2003).
21. Bescond M, *et al.* Thermionic cooling devices based on resonant-tunneling AlGaAs/GaAs heterostructure. *J. Phys. Condens. Matter*. **30**, 064005 (2018).
