# Supplementary material for: Evaporative electron cooling in asymmetric double barrier semiconductor heterostructures
Source: Nat Commun. 2019 Oct 3;10:4504. doi: 10.1038/s41467-019-12488-9 (PMC6776518; doi:10.1038/s41467-019-12488-9)
Supplement: Supplementary file 2 — Peer Review File [file 41467_2019_12488_MOESM2_ESM.pdf]

Reviewers' comments:

Reviewer #1 (Remarks to the Author):

The manuscript from Ju et al. describes the fabrication of LEDs and displays by direct pen writing of conductive and fluorescent inks. Although pen writing of these materials has been shown, the great novelty of this work is the demonstrated ability to write in a layer-by-layer fashion in order to generate functional devices such as displays. The authors described in great detail the variation in experimental conditions leading to optimum homogenous deposition of components one on top of the other. Although, as the authors themselves pointed out, still some improvement could be achieved in the performance of the final assembled LEDs, the results reported are novel and encouraging for the development of flexible displays.
I would therefore recommend this manuscript for publication after authors comment on the following points: 1) The direct pen writing showed here is simple and low cost and can achieve great control of material deposition. However, I'm not sure the method would be able to compete with other existing techniques for the generation of large area displays. 2)Have the authors tested other RGB materials alternative to QDs?. Do they see any obstacles in use of polymers or nanostructured polymer materials?. 3) Were the pens used empty of ink or were they emptied and washed prior use?

Reviewer #2 (Remarks to the Author):

SUMMARY & ANALYSIS
The paper describes the first hand-drawn, color, electroluminescent, quantum dot (QD) display. Prior work has reduced the complexity of QD display frabrication from a cleanroom process, to a printed process (especially Ref. 4). This work further reduces the barrier to entry for QD display fabrication by replacing printing with a hand-drawn process while preserving and extending the ability to fabricate on a wide variety of stiff and flexible substrates. The results are good, considering the fabrication method. The artifacts adequately establish the feasibility of hand-drawn quantum dot displays, especially Figure 7.

COMMENTS
Abstract:
What is the significance of 'boardless' in this context? How is this display boardless when it requires a substrate (e.g. glass and plastic per Figure 6)?
As a primary motivation, diversifying display form factors seems weak without an example to separate it from the previous printing work of Ref 4.
What are 'frame' manufacturing techniques?
Introduction:
How do flexible, transparent and holographic displays address the problem of fixed rectangular image shape?
Page 3
The claim that this is the first fully pen-drawn display is both significant and novel for QD displays. The creation of a display that is constructed entirely on a bench and on a wide variety of substrates has been previously reported both for QDs and on other electroluminescent platforms. EL (TFEL) is an example of a display technology that can be screen-printed, with multiple colors, on a variety of materials (wood, plastic, leather). See reference below:
Olberding, Simon, Michael Wessely, and Jürgen Steimle. "PrintScreen: fabricating highly customizable thin-film touch-displays." Proceedings of the 27th annual ACM symposium on User interface software and technology. ACM, 2014.
Perhaps the authors should acknowledge TFEL and screen-printed displays. QDs may have a number of significant advantages (lower drive voltage, higher efficiency) but it seems appropriate to acknowledge these displays as they have shared many of the goals set out in this paper and

they are present in many of the target applications mentioned in the introduction.
Page 5
How sensitive are the display characteristics to the parameters listed here such as pen speed, material thickness, and uniformity? Can we reasonably expect a hand-drawn display to produce results similar to the carefully controlled machine-drawn results described? Was the work in Figure 7 the result of multiple attempts with these parameters varied? I feel that this may warrant some discussion as it is the contribution of this paper which diverges most significantly from previous work.
Page 14
The first sentence of the last paragraph is not very precise. What is meant by the words, ‘actual device.’
References:
The references could include TFEL as noted above.
Figures:
The figures are effective artifacts and the photos appear to be free of inappropriate modification.

Reviewer #3 (Remarks to the Author):

This study offers an interesting inexpensive approach to customizing the formation of simple devices using pen drawing. The process and concept are minimally described - a number of general statements and claims are made that need justification and in some cases, an indication of values and the variance observed or expected.

Lines 79-81: What is the range of line widths that can be readily generated? Does this require pens with different tips? What is the variance or reproducibility in line width for each tip? How does this affect device properties such as luminance of the QD-LEDs?

Lines 99-102: What is a "suitable temperature"? Film properties will vary with heating temperature and probably heating time, so this statement gives no useful information.

Lines 151-154: The process apparently balances convective flow and capillary flow. How controllable and reproducible is this effect? The terms uniformity (line 155) and uniform (line 166) are used, but these terms have no meaning. How reproducible are the values of sheet resistance and transparency? Does this depend upon the air flow rate, humidity, room temperature, etc.? What variance is observed?

Lines 283-285: What does "somewhat lower" mean?

Lines 362-366: After depositing 300 nm of SiO_2 onto paper, the surface is essentially that of glass. What is the purpose of using paper substrates in this situation (I presume it is a cost issue, but the deposition adds cost also). Does the paper maintain its flexibility after deposition? Does the glass crack upon bending? Is the substrate curved after film deposition due to differences in thermal expansion coefficient?

Nearly all figures in the manuscript and in Supplementary Information will be very difficult to read when reduced to fit in journal columns. Text and numbers must be MUCH larger, especially on Figs. 2-6 and Figs. S2-S6.

We thank all reviewers for their time and constructive comments on our manuscript. We have implemented their comments and suggestions and wish to submit a revised version of the manuscript for further consideration in the journal. Changes in the initial version of the manuscript are either highlighted for added sentences or strikethrough for deleted sentences in the revised version. In the following, we provide a point-by-point response to all questions raised by the referees explaining how we have addressed each of the reviewers' comments.

Reviewer #2

I have read and review the manuscript entitled "Evaporative electron cooling in asymmetric double barrier semiconductor heterostructures" and the supplementary material. I consider that the experimental and theoretically report that the authors do complies with all the scientific rigor and detail in such a way that another laboratory that has the same infrastructure can repeat and obtain similar properties of AlGaAs/GaAs asymmetric double barrier heterostructures. The authors have shown that electrons in the quantum well are refrigerated by the evaporative cooling process. Electron temperatures in the quantum well and in the electrodes were determined by photoluminescence measurements. The authors have found that, when operated at room temperature, critic temperature in the quantum well decreases down to 250 K with increasing the bias voltage up to the maximum resonant tunneling condition. The experimental results have been well explained by quantum transport theory. The discuss the interplay between the electron and lattice cooling in the quantum well are made. Found that the lattice system is refrigerated by the thermionic cooling process and its coefficient of performance is in the order of 5-20%. I think that the manuscript "Evaporative electron cooling in asymmetric double barrier semiconductor heterostructures" can be considered for publication in Nature Communications in the present form.

Authors' response #2: We thank the reviewer for his appreciation and the positive comments on our manuscript without any concern.

Reviewer #3

The authors demonstrate a thermionic cooler based on vertical electron transport in an AlGaAs heterostructure. The key idea has been described/simulated in a recent paper by the same authors, JAP 30, 064005 (2018). The experiment provides an interesting demonstration of evaporative cooling at room temperature. I believe it can meet the interest of a wide audience and it might be suitable for publication on NC. Few comments should be addressed before publication:

Authors' response #3: We thank reviewer #3 for his/her positive comments as a whole.

1) *Intro part. The authors state that conventional Peltier technology is "not compatible with standard semiconductor fabrication processes". I can agree with this statement. However, also AlGaAs is not really completely compatible with current Si-based technology either. Can a monolithic cooler can be envisioned? Alternatively, could this architecture be realized, for instance, in a Si-based heterostructure?*
*I am also not fully sure about the statement "the thermoelectric process is continually interrupted by phonon scattering". I don't completely get what "interrupted" means here. Also, ruling out phonon drag, the main (detrimental) role of phonons should just be to provide a parallel heat conduction path. However, isn't this going to occur in *any* solid-state device, including those based on (solid-state) thermionic emission?!*

Authors' response #3-1: We agree with the reviewer #3 that it is not easy to incorporate the present III-V-based heterostructure refrigerators into the Si-based LSI technology, although a significant progress has recently been made in heterogeneous III-V/Si integration. By "not compatible…" in the original manuscript, we meant that the commonly used thermoelectric materials such as bismuth tellurides are much less compatible with the conventional device technologies than III-V compounds. We would like to point out that the present device concept of thermionic cooling can also be realized by using the SiGe-based heterostructure systems. Furthermore, the present heterostructure cooling device can be more naturally integrated with optoelectronic devices such as semiconductor lasers.

We agree that the expression *"the thermoelectric process is continually interrupted by phonon scattering"* might be a bit clumsy. By "interrupted" we mean that the cooling efficiency of a thermoelectric device is degraded by Joule heating through electron scattering, which does not occur in ideal thermionic cooling structures.

For the sake of clarity, we replaced the sentence with: (p.2, line 14)
"The most commonly used solid-state refrigeration is based on the thermoelectric Peltier effect.[12] *In the thermoelectric regime, electrons frequently experience scattering, leading to degradation in the thermoelectric power factor $S^2\sigma$. Here, S and σ are the Seebeck coefficient and the electrical conductivity, respectively. Furthermore, the materials used to obtain efficient Peltier effect such as BiTe are not compatible with the standard semiconductor fabrication processes."*

2) *The device structure reported in Fig.1a is not completely satisfactory. First of all, there is a missing 5nm undoped GaAs layer (what is its purpose, exactly? maybe a comment on this would help) and I think it would also be good to explicitly indicate the GaAs substrate in the figure. In addition, I think it would also be useful to include a complete sketch of the device, with the ohmics (size, position etc), the thin NiCr layer (whose role probably deserves few additional comments: for instance, why is it so much smaller than the mesa?) etc…*

Authors' response #3-2: In the revised version of the manuscript, we have updated the band diagram of the asymmetric double barrier heterostructure by adding the 5 nm-undoped GaAs layer (Figure 1a). This 5 nm-thick undoped GaAs layer is used as a spacer layer to prevent the segregation of Si dopant atoms into the undoped AlGaAs emitter barrier and the GaAs quantum well (QW).

Moreover, following the reviewer suggestion, we have added a new figure describing the sample in the Supplementary Information section (Supplementary Figure 1).

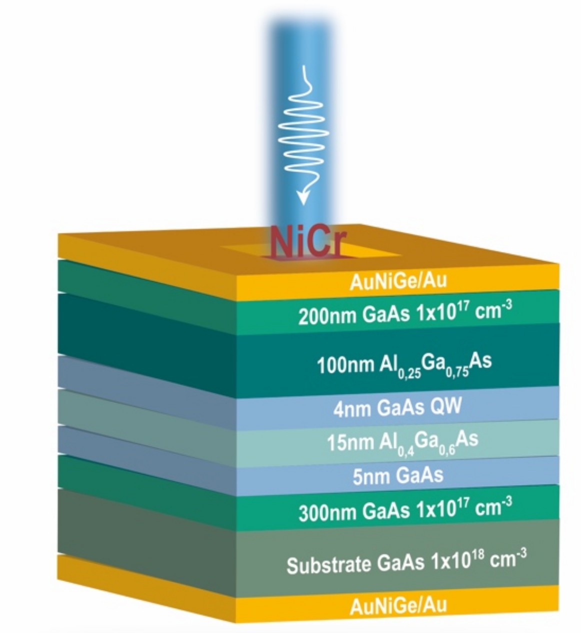

Supplementary Figure 1: Schematic illustration of the sample used in this study.

We have also added the following sentence in the Supplementary Information section:
"The Supplementary Fig. 1 schematically represents the sample used in this work. The growth sequence is described in the main text. We note that the 5 nm-thick undoped GaAs layer below the emitter barrier is inserted to prevent the diffusion of Si donors into the thin $Al_{0.4}Ga_{0.6}As$ emitter barrier due to the segregation effect. The fabrication process is described in the main text."

3) *The device architecture includes a thicker opaque barrier and a thinner one which is dominated by tunnel coupling. Clearly, the transparency of the thin barrier is crucial for the device operation since it thermally decouples the QW from the emitter electron systems. The authors should clarify – at least theoretically – how important is the tuning of the injection barrier for the correct operation of the device.*

Authors' response #3-3:
We thank the reviewer for asking this very important question.
The role of the thin emitter barrier is indeed to inject "cold" electrons by resonant tunneling into the quantum well. As shown theoretically (please see Fig. 4 of Ref. [16] of the SI), the device correctly

operates even when the emitter barrier is as thin as 2 nm, as assumed in the original paper by Chao (Ref. 26). However, for such a thin barrier thickness the heat insulation between the electrons in the QW and those of the emitter becomes worse and the electron cooling is degraded.

Moreover, when the emitter barrier was 2-nm-thick, the PL from the QW could not be resolved from that from the electrodes, because the electronic states in the QW was significantly hybridized with the states in the emitter electrode. Therefore, a thicker emitter barrier (15 nm in the present case) was used to quantum mechanically decouple the electronic states in the QW from the states in the emitter contact. This comment has been added in the supplementary information.

In the revised version, the following sentence has been added in the main text: (p. 3, line 20)
"We used a rather thick emitter barrier (15 nm) to quantum-mechanically decouple the electronic states in the QW from those in the emitter electrode and to separate the photoluminescence (PL) in the QW from that in the electrodes. Thinner barrier would also degrade the heat insulation between the electrons in the QW and those of the emitter, leading to a reduction of the electron cooling."

4) *About PL data. The authors indicate the large peak at 1.4eV as the "electrode". What about the PL from the substrate? I am also wondering about the effect of hot electrons escaping from the QW and landing in the collector. Shouldn't they heat up the collector? Is there any evidence of such a heating? Or is maybe the collector so well-anchored to the bath temperature that the effect can be neglected? More on this in the next point.*

Authors' response #3-4:
First of all, we want to make it clear that, by "the electrodes", we mean both the top (collector) and the bottom (emitter) electrodes. As the reviewer pointed out, the electrode PL originates not only from the top electrode but also from the bottom electrode. The real substrate is located too deep for this excitation photon energy (the penetration depth in GaAs is only ~100 nm at 2.54 eV).

As the reviewer pointed out, the electrons in the collector electrode are indeed heated up by the landing of hot electrons that escape the QW. However, experimentally, we did not observe a significant change in the electron temperature in the collector, as seen Fig. 3d. This is reasonable because the heat released by the hot electrons are quickly redistributed and shared among very many electrons in the collector electrode. Since the electron density in the QW is in the order of 10^{14} cm^{-3} in the resonant tunneling condition, while that in the collector electrode is 10^{17} cm^{-3}, the increase in the electron temperature in the collector is expected to be very small (< 0.1 K). Furthermore, the surface metal electrode also helps good thermal anchoring of electrons in the collector electrode.

The following sentence has been then added in the revised version of the paper: (p. 6, line 3 from the bottom)

"We note that electrons in the collector are heated up by the landing of hot electrons that escape from the QW. However, since the heat released by the hot electrons are very quickly redistributed and shared among very many electrons in the collector electrode, the increase in the electron temperature in the collector is expected to be much smaller (< 0.1 K). Furthermore, the surface metal electrode also helps good thermal anchoring of electrons in the collector electrode."

5) *The model is already described in the JAP2018 and in the SI, however it would be nice to add some info about it. For instance, it would be nice to understand the origin of the error bars (and the scattering of their values) in Fig.3d: at the moment they are not completely clear to me. Also, was the choice kappa_QW = 4W/mK used as a free fitting parameter to reproduce the experimental data? If so I think this should be explicitly mentioned. So far, the choice is not very much motivated (quantitatively, I mean). The boundary conditions also deserve some comment: both the contacts are assumed to be at thermal equilibrium with the bath at 300K. Is this due to a large and efficient electronic heat conduction in the doped GaAs regions? Can the authors argue that this assumption correctly describes the behavior of the real device? Some nonnegligible power is being dissipated in terms of hot electrons entering the collector; since the authors put there a boundary condition of thermal equilibrium, I wonder if/how dissipation is taken into account in the modelling.*

Authors' response #3-5:

We thank the reviewer for raising these important points.

- The calculation of the electron temperature in the QW is performed based on the virtual probe approach, which is described in the SI. This approach allows us to calculate the local electron temperature in nanostructures operating in a non-equilibrium regime. The main idea is to enforce the simultaneous cancellation of the electron charge and energy currents between the device and the probe by modifying the electron temperature and the electrochemical potential in order to get the probe in local thermal equilibrium with the nanostructure.

In the PL measurements, we basically measure the energy distribution of electrons that relax their energy in the QW and recombine with holes. In other words, the PL measurements do not see electrons that ballistically fly over the collector barrier. In the virtual probe approach, we have then limited the upper energy integration interval to an energy at the top of the collector barrier in the electron charge and energy current calculations, in order to remain as faithful as possible to the experimental conditions. Since the calculated electron temperature depends on the choice of this upper integration limit, we have selected four values for this upper integration limit and have taken an average of the calculated temperatures. Since the number of hot electrons transmitted ballistically from the emitter to the collector increases with increasing bias voltage, the error bars are enhanced for higher applied biases.

In order to clarify this point, we have modified the SI as follows:

"By enforcing the simultaneous cancellation of the electron charge and energy currents between the device and the probe, we obtain a system of two coupled nonlinear equations with the unknowns μ_j *and* T_j^e*:*

$$\Delta J(j) = \int_{-\infty}^{E_{Lim}} \Sigma^{>}(j;E)\, G^{<}(j,j;E) dE - \int_{-\infty}^{E_{Lim}} G^{>}(j,j;E)\Sigma^{<}(j;E)\, dE = 0, \qquad (16)$$

$$\Delta J^{E}(j) = \int_{-\infty}^{E_{Lim}} E\, \Sigma^{>}(j;E)\, G^{<}(j,j;E) dE - \int_{-\infty}^{E_{Lim}} E\, G^{>}(j,j;E)\Sigma^{<}(j;E)\, dE = 0. \quad (17)$$

The upper energy integration limit E_{Lim} *results from the fact that PL measures the energy distribution of electrons that relax their energy in the QW and recombine with holes. In other words, the PL measurements do not see electrons that ballistically fly over the collector barrier.*
In order to stay as faithful as possible to the experimental conditions, we therefore theoretically consider electrons whose energy is lower than the top of the emitter barrier. Since the electron temperature depends on E_{Lim}*, we used four different* E_{Lim} *and took an average of the obtained values. Moreover, the quantity of hot electrons which are transmitted from the emitter towards the collector by direct tunneling increases with the applied bias. The choice of* E_{Lim} *is then more impacting the electron temperature at high biases. This is the reason why we have larger error bars for higher biases in Fig.3-d of the manuscript."*

- The choice of κ_{QW} = 4W/Km is not a fitting parameter which was used to reproduce the data. This value is motivated by experimental and theoretical studies (please see, for instance, M. N. Luckyanova, J. A. Johnson, A. A. Maznev, J. Garg, A. Jandl, M. T. Bulsara, E. A. Fitzgerald, K. A. Nelson, and G. Chen, *Nano Lett.* **13**, 3973, (2013) - DOI: 10.1021/nl4001162), showing that the cross-plane thermal conductivity in GaAs/AlAs superlattices is strongly reduced by increased diffusive phonon scattering at the heterointerfaces. Indeed, they reported that the thermal conductivity can be as small as a few W/Km. The choice of 4W/Km in the present work is based on such a report.

In the supplementary information (p. 5, line 9), we explicitly mentioned that "… except in the QW region, where it is set to be 4 W/Km to take into account the thermal resistance associated with heterointerfaces [M. N. Luckyanova, J. A. Johnson, A. A. Maznev, J. Garg, A. Jandl, M. T. Bulsara, E. A. Fitzgerald, K. A. Nelson, and G. Chen, *Nano Lett.* **13**, 3973, (2013) - DOI: 10.1021/nl4001162]. A small deviation of the value has no significant impact in the calculated electron temperature."

- The boundary conditions in our calculation assume the contacts to be at 300 K. This assumption indeed corresponds to massive contacts in which all the hot electrons are thermalized. In the present sample structure, a long collector region (200 nm-thick) allows electrons to be entirely thermalized. In this configuration, the heat power density generated by the electron-phonon interactions Q_j (Eq. 12 of the Supplementary Information) integrated over the whole device must be equal to the applied power density, $P_{Elec} = J \times V$. In the calculations, this point has been verified systematically for each applied bias. We also note that reducing the collector length to 100 nm does not affect the calculated

electron temperature in the quantum well.

These aspects have been added in the revised Supplementary Information section (p. 7):
"We note that the assumption of the contacts at 300K corresponds to massive contacts with sufficient electron thermalization. In the present study, we considered a long enough collector region (200 nm) to allow electrons to be entirely thermalized. In this configuration, the heat power density Q_j integrated over the whole device must be equal to the applied power density, $P_{Elec} = J \times V$. In the calculations, this point has been verified systematically for each applied bias. We should also note that reducing the collector length to 100 nm does not affect the calculated electron temperatures in the quantum well."

6) *Some statistical information about the study should be given. Was the study performed on a single device, on many ones? If so, on how many?*

Authors' response #3-6: We tried different sample structures with various structural parameters, in particular, the thickness of the emitter barrier as well as the thickness of the QW. The most crucial factor was to separate the PL from the QW and that from the electrodes. When the emitter barrier was increased to 15 nm, we could decouple the QW states from the states in the electrode and resolve two PL components. We grew 3 wafers with a 15-nm-thick emitter barrier and observed the cooling behavior in all of them. We reported in this manuscript the results for the best parameterized structure.

We have addressed all the remarks and questions of the reviewers. We believe that this revised version of the paper is now suitable for publication in Nature Communications.

Yours sincerely,

Marc Bescond on behalf of all the co-authors.

Reviewers' comments:

Reviewer #3 (Remarks to the Author):

The authors have satisfactorily replied to all my comments and the paper is suitable for publication on NC in its present form.

Reviewer #4 (Remarks to the Author):

The manuscript proposes a novel cooling concept based on evaporative cooling of electrons with a heterostructure device. The physical mechanism is well described and also experimentally demonstrated. While this concept is interesting and seems novel, I still have some concerns over its practical implications. If the authors can thoroughly address the following points I would recommend publication in Nature Communications.

1. The authors mentioned that a higher doping density in the emitter should enhance lattice cooling of their device which currently only allows for a few mK temperature drop in theory. Can the authors estimate what doping level they need to achieve a practically useful temperature difference? And what will be the COP at that point?

2. Can the authors quantify the maximum heat flux that can pass through this cooling device? Or what needs to be done to achieve heat dissipation ~ 100 W/cm2 (which is used as a target in the introduction)? And will the COP depend on the heat flux?

3. Can the authors detail how the error bars are determined in Fig. 2d and Fig. 3d?

Reviewer #5 (Remarks to the Author):

The authors have successfully revised the manuscript to address the comments by Referee 3, and it appears that both Referees 2 and 3 are in favor of publishing the manuscript after the revision.

I have read the paper with interest and find it suitable for publication in Nature Communications, but I would like to ask the authors to address three points before the manuscript is published:

1) On page 7, the authors correctly state that in polar semiconductors such as GaAs the electron-phonon interactions are dominated by the polar optical phonons. They cite ref. 36, which is a paper on GaN; it would be useful to add a citation to another paper on GaAs by the same authors (Zhou et al., Phys. Rev. B 94, 201201, 2016) , which clearly shows that in GaAs the main contribution to scattering at low energy is from the polar optical phonons.

2) The main evidence for the 50K electron cooling is the PL measurement in Fig. 3c, and in particular the fitting of the PL data to the models in Eqs. 1-2 on page 5. However, as the authors state, these models for the PL intensity are approximate. One point the paper should address more carefully is whether these models have been used before to extract the electron temperature, whether their accuracy is known / has been validated, and what are the margins of error of extracting an electron temperature this way. This point seems quite crucial since the main finding depends critically on it.

3) The electron-phonon self-energy expression, Eq. 5 on page 4 of the Supplementary Material, appears to be derived in ref. 7 of the supplementary material. This reference is a mysterious private communication from year 2108 (!). It would be great to update the year in this reference

and to say a few more words about how this formula and approach have been derived.

The manuscript can be published after these changes have been made.

First of all, we thank all the reviewers for their time and constructive comments on our manuscript. We have implemented their comments and suggestions and wish to re-submit a revised version of the manuscript for further consideration in the journal. Changes in the previous version of the manuscript are highlighted in yellow in the revised version. In the following, we provide a point-by-point response to all the questions raised by the referees, explaining how we have addressed each of the reviewers' comments.

Reviewer #3

The authors have satisfactorily replied to all my comments and the paper is suitable for publication on NC in its present form.

Authors' response #3: We thank the reviewer for his/her satisfaction and appreciation on our manuscript.

Reviewer #4

The manuscript proposes a novel cooling concept based on evaporative cooling of electrons with a heterostructure device. The physical mechanism is well described and also experimentally demonstrated. While this concept is interesting and seems novel, I still have some concerns over its practical implications. If the authors can thoroughly address the following points I would recommend publication in Nature Communications.

Authors' response #4: We thank reviewer #4 for his/her positive comments as a whole and in the following we provide our response to all his/her comments.

1) *The authors mentioned that a higher doping density in the emitter should enhance lattice cooling of their device which currently only allows for a few mK temperature drop in theory. Can the authors estimate what doping level they need to achieve a practically useful temperature difference? And what will be the COP at that point?*

2) *Can the authors quantify the maximum heat flux that can pass through this cooling device? Or what needs to be done to achieve heat dissipation ~ 100 W/cm^2 (which is used as a target in the introduction)? And will the COP depend on the heat flux?*

Authors' response #4-1 & #4-2: The main purpose of this work is to show, in a proof-of-concept manner, that the evaporative cooling effect can efficiently refrigerate electrons at room temperature

in semiconductor heterostructures. The sample used in this work was, therefore, optimized for detecting the electron cooling, but not for maximizing the cooling power. In particular, we used a thick (15 nm-thick) emitter barrier and a low doping concentration in the electrode regions to clearly separate the PL peak of the QW from that of the electrodes (as mentioned in the manuscript).

Lattice refrigeration is also discussed in this paper through quantum transport simulations. Although a comprehensive lattice cooling study is beyond the scope of the present paper, we have shown that the considered device structure can also have a favorable impact on the lattice temperature. Lattice refrigeration requires a higher doping density in the emitter electrode and a thinner emitter barrier in order to increase the current density and, as a consequence, the cooling power density. Theoretical investigations reported in ref [40] of the revised manuscript have shown that a structure with a higher doping level (~10^{18} cm^{-3}) in the emitter and a thinner emitter barrier (~2.4 nm) can reach a COP greater than 100% at a bias voltage $V = 0.1$ V with a cooling power density as large as 2×10^2 W/cm^2. Note that the COP in the QW can be larger than 100 % by transferring heat from the QW to other regions of the structure. However, since it would become much more difficult to characterize the electron and lattice temperatures in the QW in such a high current-density structure, the demonstration of the lattice cooling effect is going to be our future work.

For the sake of clarity, we have added on p. 8 of the revised manuscript the following sentences:
"… an efficient lattice cooling requires a higher current density in order to realize a cooling power density of the order of 10^3 W/cm^{-3} in the QW. This value can be obtained by increasing the doping density in the emitter electrode and by reducing the thickness of the emitter barrier. Theoretical investigations reported in Ref. [40] have shown that a structure with a higher doping level of 10^{18} cm^{-3} in the emitter and a thinner emitter barrier (2.4 nm) can achieve a maximum COP above 100% at low bias voltages ($V \sim 0.1$ V) and realize a cooling power density of 2×10^2 W/cm^{-2}, suggesting that fully optimized structures will be able to locally refrigerate the lattice by several degrees °C under typical operating conditions."

3) *Can the authors detail how the error bars are determined in Fig. 2d and Fig. 3d?*

Authors' response #4-3:
The activation energies (Fig. 2d) were determined from the slopes of the log(J/T^2) *vs* $1/T$ plots at high temperatures (Fig. 2c), where we observe the thermal activation behavior. The slopes were obtained by the least square fitting. The errors bars shown in Fig. 2d correspond to the standard deviation.

The error bars for determining the electron temperatures in the QW and the electrodes (Fig. 3d) arise from an uncertainty that results from the background subtraction in the PL spectra. We subtracted the background counts from the PL spectra measured in the energy range from 1.3 eV to 1.7 eV. We estimate the error in the obtained T_e which may arise from the background subtraction is ~ 3%.

The origin of the error bars for the calculated electron temperatures in the QW (blue and pink triangles in Fig. 3d) has been already described in the SI. The error bars originate from the upper energy integration limit in the integrals of Eqs. (16) and (17) of the SI. The calculation of the electron temperature in the QW was performed by using the virtual probe approach. This approach allows us to calculate the local electron temperature in nanostructures that operate in a non-equilibrium regime. The main idea is to enforce the simultaneous cancellation of the electron charge and energy currents between the device and the probe by modifying the electron temperature and the electrochemical potential in order to let the probe be in a local thermal equilibrium with the nanostructure.

In the PL measurements, we basically measure the energy distribution of electrons that relax their energy in the QW and recombine with holes. In other words, the PL measurements do not see electrons that ballistically fly over the collector barrier. Therefore, in the virtual probe approach, we have limited the upper energy integration interval to the energy at the top of the collector barrier, in order to remain as faithful as possible to the experimental conditions. Since the calculated electron temperature depends on the choice of this upper integration limit, we have selected four values for this upper integration limit and have taken an average of the calculated temperatures. Since the number of hot electrons transmitted ballistically from the emitter to the collector increases with increasing bias voltage, the error bars are enhanced for higher applied biases. Please refer to the SI part for more details.

Reviewer #5

The authors have successfully revised the manuscript to address the comments by Referee 3, and it appears that both Referees 2 and 3 are in favor of publishing the manuscript after the revision.
I have read the paper with interest and find it suitable for publication in Nature Communications, but I would like to ask the authors to address three points before the manuscript is published:

Authors' response #5: We thank reviewer #5 for his/her positive comments as a whole.

1) *On page 7, the authors correctly state that in polar semiconductors such as GaAs the electron-phonon interactions are dominated by the polar optical phonons. They cite ref. 36, which is a paper on GaN; it would be useful to add a citation to another paper on GaAs by the same authors (Zhou et al., Phys. Rev. B 94, 201201, 2016), which clearly shows that in GaAs the main contribution to scattering at low energy is from the polar optical phonons.*

Authors' response #5-1: We thank the referee for his/her suggestion. The reference has been included in the revised version of the manuscript as Ref. [37].

2) *The main evidence for the 50K electron cooling is the PL measurement in Fig. 3c, and in particular*

the fitting of the PL data to the models in Eqs. 1-2 on page 5. However, as the authors state, these models for the PL intensity are approximate. One point the paper should address more carefully is whether these models have been used before to extract the electron temperature, whether their accuracy is known / has been validated, and what are the margins of error of extracting an electron temperature this way. This point seems quite crucial since the main finding depends critically on it.

Authors' response #5-2: The determination of electron temperatures from the fit of the high energy tail of the PL spectra, assuming the Maxwell-Boltzmann distribution, has been well established and has often been used particularly for GaAs/AlGaAs heterostructures (please see for instance refs [31-33] of the manuscript). However, we agree with the reviewer that the calculation of the electron temperature directly from the slope of high-energy tail of PL spectra relies upon several approximations:

- First of all, the slope of a PL spectrum reflects both the electron and hole temperatures. However, holes, which have a heavier effective mass, are supposed to have a temperature closer to the lattice temperature, while electrons are supposed to be in a non-equilibrium state at a different temperature given by the slope of the PL spectra [J. Shah, C. Lin, R.F. Leheny, A.E. DiGiovanni, *Solid State Commun.* **18**, 487 (1976)]. A good hint to assess this statement is given by the ratio $\alpha = m_e/(m_e+m_h)$. The smaller is α, the closer is the slope of the PL to the electron temperature. In GaAs, m_e=0.67m_0 and m_h=0.45m_0, leading to α=0.13. It has been shown that, when α=0.05, the electron temperature calculated from the slope of the PL spectra is equal to the electron temperature extracted from the general expression of Planck's law with two temperatures with an accuracy of 0.05% (please see F. Gibelli, L. Lombez, et J.-F. Guillemoles, *Phys. B Condens. Matter* **498**, 7-14 (2016)). Although α for GaAs is not as small as 0.05, we can still safely assume that the impact of the hole component on slope of the PL spectra is negligible and that this PL technique provides a good assessment of the electron temperature.

- The second approximation comes from the fact that we assumed that the PL originates from the band-to-band transition and we neglected the excitonic emission effects. However, since exciton binding energy in GaAs is of the order of 5 meV (see S.Tarucha, H.Okamoto, Y.Iwasa, N.Miura, *Solid State Commun.*, **52**, 815-819 (1984)), we can safely neglect the excitonic effect in PL measurements at room temperature.

- The third main approximation lies in the energy-dependence of the absorption coefficient $A(E)$. Indeed, a more "rigorous" description of the PL spectrum includes the absorption coefficient as follows (see Y. P. Yu, M. Cardona, Fundamentals of Semiconductors; Springer: Berlin, 2005; pp 346−354):

$$I_{PL}(E) \propto A(E)e^{-\frac{(E-E_g)}{k_bT}}.$$

Approximating the high-energy PL tail by a "simple" exponential assumes that the absorption coefficient is energy-independent. Study from Ihara and co-workers have demonstrated that such assumption is indeed accurate in GaAs QWs at temperatures from 20 K to 220K (please see T. Ihara, S. Maruyama, M. Yoshita, H. Akiyama, L. N. Pfeiffer and, K. W. West, *Phys. Rev. B* **80** 033307 (2009)). This result should remain valid at room temperature, validating the use of our technique to prove the electron cooling phenomena in the GaAs QW.

In view of these explanations, the determination of the electron temperature based on the high-energy slope of the PL spectra is reliable to demonstrate the electron cooling by several tens of kelvins.

In the revised version of the manuscript, these considerations have been included on page 2 in the Supplementary Information section.

3) *The electron-phonon self-energy expression, Eq. 5 on page 4 of the Supplementary Material, appears to be derived in ref. 7 of the supplementary material. This reference is a mysterious private communication from year 2108 (!). It would be great to update the year in this reference and to say a few more words about how this formula and approach have been derived.*

Authors' response #5-3: We thank the referee for highlighting this mistake. We corrected and updated the reference 7 of the SI (which now appear as reference [15] in the SI):

M. Moussavou, M. Lannoo, N. Cavassilas, D. Logoteta and M. Bescond, "Physically based diagonal treatment of polar optical phonon self-energy: performance assessment of III-V double-gate transistors," *Phys. Rev. Appl.* **10**, 064023 (2018).

We have addressed all the remarks and questions of the reviewers. We believe that this re-revised version of the paper is now suitable for publication in Nature Communications.

Yours sincerely,

Marc Bescond on behalf of all the co-authors.

REVIEWERS' COMMENTS:

Reviewer #4 (Remarks to the Author):

I believe the authors have done an excellent job of answering my questions (and for that matter those of the other referees). I believe the manuscript is now suitable for publication in Nat. Comm.

Reviewer #5 (Remarks to the Author):

I am happy with the authors' revision of the manuscript and recommend it for publication in its current form.

Date: August the 22nd, 2019

First of all, we thank all the reviewers for their time and constructive comments on our manuscript. In the following, we provide a point-by-point response to all the questions raised by the referees.

In the following, we provide a point-by-point response to the referees comments.

Reviewer #4

I believe the authors have done an excellent job of answering my questions (and for that matter those of the other referees). I believe the manuscript is now suitable for publication in Nat. Comm.

Authors' response #4: We thank the reviewer for his/her satisfaction and appreciation on our revised manuscript.

Reviewer #5

I am happy with the authors' revision of the manuscript and recommend it for publication in its current form.

Authors' response #5: We thank reviewer #4 for his/her positive recommendation.

Yours sincerely,

Marc Bescond on behalf of all the co-authors.
